# Lipid Raft Is Required for PSGL-1 Ligation Induced HL-60 Cell Adhesion on ICAM-1

**DOI:** 10.1371/journal.pone.0081807

**Published:** 2013-12-03

**Authors:** Tingshuang Xu, Wenai Liu, Jixian Luo, Chunfeng Li, Xueqing Ba, Khamal Kwesi Ampah, Xiaoguang Wang, Yong Jiang, Xianlu Zeng

**Affiliations:** 1 Institute of Genetics and Cytology, Northeast Normal University, Changchun, Jilin Province, China; 2 Department of Bioscience, Shanxi University, Taiyuan, China; 3 Department of Bioscience, Changchun Teachers College, Changchun, China; 4 Department of Pathophysiology, Southern Medical University, Guangzhou, China; Northwestern University, United States of America

## Abstract

P-selectin glycoprotein ligand-1 (PSGL-1) and integrins are adhesion molecules that play critical roles in host defense and innate immunity. PSGL-1 mediates leukocyte rolling and primes leukocytes for integrin-mediated adhesion. However, the mechanism that PSGL-1 as a rolling receptor in regulating integrin activation has not been well characterized. Here, we investigate the function of lipid raft in regulating PSGL-1 induced β2 integrin-mediated HL-60 cells adhesion. PSGL-1 ligation with antibody enhances the β2 integrin activation and β2 integrin-dependent adhesion to ICAM-1. Importantly, with the treatment of methyl-β-cyclodextrin (MβCD), we confirm the role of lipid raft in regulating the activation of β2 integrin. Furthermore, we find that the protein level of PSGL-1 decreased in raft fractions in MβCD treated cells. PSGL-1 ligation induces the recruitment of spleen tyrosine kinase (Syk), a tyrosine kinase and Vav1 (the pivotal downstream effector of Syk signaling pathway involved in cytoskeleton regulation) to lipid raft. Inhibition of Syk activity with pharmacologic inhibitor strongly reduces HL-60 cells adhesion, implicating Syk is crucial for PSGL-1 mediated β2 integrin activation. Taken together, we report that ligation of PSGL-1 on HL-60 cells activates β2 integrin, for which lipid raft integrity and Syk activation are responsible. These ﬁndings have shed new light on the mechanisms that connect leukocyte initial rolling with subsequent adhesion.

## Introduction

Neutrophils are essential component of the innate immune system. The recruitment of neutrophils to a site of infection or tissue injury entails a cascade of cellular adhesive events, including tethering, rolling, adhesion, diapedesis, transmigration and chemotaxis [1-3]. P-selectin glycoprotein ligand 1 (PSGL-1) is expressed by most leukocytes and functions as a common ligand for the three selectins [4-6]. Early in inflammation, PSGL-1 binds to P- or E-selectin expressed on activated endothelium, and mediates leukocyte rolling on endothelium. Subsequently, integrins are activated and mediate the cell arrest and stable adhesion to endothelium under shear flow conditions [2,7]. However, the two steps seem not be separate. PSGL-1/selectin binding appears to be involved in the activation of integrin in a gradual manner [8,9]. Therefore, investigating the molecular mechanism underlying the PSGL-1-induced signals and integrin activation is of great importance for understanding the relationship between the initial rolling and subsequent adhesion.

In addition to its direct role in the capture of leukocytes from the bloodstream, PSGL-1 also functions as a signal transduction receptor and initiates a series of intracellular signal events during leukocytes activation, including secretion of cytokines, activation of integrins, transcriptional activation [10-12], especially the activation of kinases, such as MAPK, Syk, c-Abl and Src [9,11-13]. Although these events are typically associated with integrin activation, the relationship between PSGL-1-induced signals and β2 integrin activation is not well defined. 

Firm adhesion of leukocytes to the endothelium is mediated by integrins, a large family of transmembrane heterodimeric adhesion receptors expressed on the cell surface of eukaryotic cells. Neutrophils express β2 integrins, αLβ2 (CD11a/CD18, LFA-1) and αMβ2 (CD11b/CD18, Mac-1), which mediate leukocytes arresting to inflamed microvessels [14,15]. To efficiently bind ligands, integrin must be activated. Integrin activation is induced by either a conformational change within each receptor, which increases apparent afﬁnity for ligand, or integrin clustering, which enhances avidity for ligand [16,17]. Accumulating evidence demonstrates that clustering of LFA-1 receptors in the plane of the membrane not only strengthens cell–cell adhesion but also is a prerequisite for LFA-1 activation and ligand binding [16]. In previous studies, the mechanisms underlying the regulation of integrin activation by rolling receptor were mainly focused on affinity changes [8,17], however the regulation of integrin clustering by PSGL-1 as well as its role in neutrophil adhesion have not been well identiﬁed.

Lipid rafts have been extensively studied as cellular signaling platforms, particularly in T cells and other leukocytes [18-21]. Lipid rafts of the membrane possess distinct structural and compositional properties that allow them to harbor some signaling proteins and exclude others [22-24]. In particular, both PSGL-1 and integrin have been found to be membrane rafts associated [25-27]. Sequestering cholesterol blocks selectin-mediated activation of Syk kinase and selectin-dependent rolling [28]. However, the role of lipid rafts in regulating integrin activation in PSGL-1 ligated leukocyte remains to be understood. 

In the present study, we used HL-60 cells as surrogates of neutrophil for our studies involving PSGL-1 ligation-induced integrin activation. Here, a novel mechanism that lipid raft controls β2 integrin activation and cell adhesion in PSGL-1 ligated HL-60 cells is well-deﬁned. In addition, we demonstrate a correlation between β2 integrin activation and lipid raft in PSGL-1 ligated HL-60 cells. Furthermore, we find that upon ligation of PSGL-1 on HL-60 cells, Syk is activated, which is consistent with the recruitment of Vav1 to the lipid raft. Our results show for the first time that integrin is associated with lipid raft under rolling receptor engagement conditions. 

## Materials and Methods

### Reagents and antibodies

LY294002 (a specific inhibitor to PI3K), Piceatannol (a specific inhibitor to Syk/ZAP-70 kinase), methyl-β-cyclodextrin (MβCD), soluble cholesterol, HRP-CtxB, FITC-conjugated Phalloidin, PY20 (anti-phosphotyrosine monoclonal antibody) and non-conjugated F(ab’)2 fragment of goat anti-mouse IgG were from Sigma-Aldrich. Chelerythrine chloride was from Enzo Life Sciences. STI571 (a specific inhibitor to nonreceptor tyrosine kinase c-Abl) was a gift from Novartis Pharma Schweiz AG (Basel, Switzerland). rhICAM-1/Fc (Recombinant chimeric human ICAM-1) was from R&D. KPL1 (the anti-PSGL-1 monoclonal antibody, mouse IgG1, sc-13535), FITC-IB4 (167-040), and mouse IgG2a (281-010) were obtained from Ancell. MEM48 (anti-CD18 mAb, mouse IgG1) was from Chemicon International. TS1/18 (the mAb against CD18, which can block the interaction of β2 integrin with ICAM-1, mouse IgG1, MA1810) was purchased from Pierce Biotechnology (Rockford, IL, USA). CD11a was from BD Biosciences. PE-antihuman CD11b was from Sungene Biotech. Alexa Fluor 488 conjugated-cholera toxin subunit B (CTXB-488) was from Invitrogen. αL/M/X/β2 (mouse anti-human β2 monoclonal antibody, recognize total β2 activation epitope), Mouse IgG1 antibodies to Vav1, Syk, flotillin-2 were purchased from Santa Cruz Biotechnology. 

### Cell stimulation

HL-60 cells were resuspended in PBS, and incubated with mIgG or KPL1 at a concentration of 10 μg/ml at 4°C for 20 min, and then ligated with 20 μg/ml F(ab’)2 of goat anti-mouse IgG at 37°C for indicated time. For inhibition experiments, cells were preincubated with MβCD, LY294002 (50 μM), Piceatannol (10 μM), STI571 (10 μM), Chelerythrine chloride (20 μM) respectively or equal volume of DMSO before addition of antibodies.

### Cell adhesion assay under static conditions

ICAM-1 and control human IgG were diluted in PBS, added to 96-well tissue culture plates (100 μl containing 5 μg/well) at 4°C overnight, and then blocked with BSA at 37°C for 2 h. After washing the plates with PBS, cells were incubated with 1 μM calcein-acetoxymethyl ester at 37°C for 30 min, and then resuspended in IMDM. To cross-link PSGL-1 with Abs, the labeled cells were incubated with 10 μg/ml anti-PSGL-1 mAb KPL-1 or control mIgG at 4°C for 20 min, washed, and then incubated with 20 μg/ml goat F(ab’)2 anti-mouse IgG at 37°C for indicated time. The cells were washed and added to triplicate wells (100 μl IMDM containing 5 × l0^4^ cells) and incubated for 20 min on ice, and then rapidly warmed at 37°C for 10 min. In some experiments, PSGL-1 ligated cells were preincubated with an anti-β2 integrin mAb TS1/18 or its isotype control mIgG before addition to the plate. The percentage of adherent cells, before and after washing, was calculated by dividing the latter value by the former. For inhibitory experiments, HL-60 cells were preincubated with STI571 (10 μM), MβCD (10mM), LY294002 (50 μM), Piceatannol (10 μM), Chelerythrine chloride (20 μM) respectively or equal volume of DMSO/PBS at 37°C for 30 min before stimulation.

### Cell adhesion assays under ﬂow conditions

Recombinant ICAM-1 or control human IgG (50 μg/ml) was immobilized on polystrene petridishes at 4°C overnight, and then blocked at room temperature with 2% human serum albumin for 30 min. HL-60 cell adhesion on ICAM-1 was measured *in vitro* using a parallel-plate ﬂow chamber (Glyco Tech, Rockville, MD). PSGL-1 in HL-60 cells was ligated with KPL-1 and a secondary antibody. The cells were resuspended at 1×10^6^ cells/ml in IMDM and perfused over the rhICAM-1-Fc monolayer via a syringe pump at a shear stress of 1 dyn/cm^2^. The interaction between HL-60 cells and ICAM-1 was visualized and recorded by an inverted microscope (Olympus Optical, Tokyo, Japan) equipped with a camera (Panasonic, Yokohama, Japan) connected to a VCR and a computer monitor. Video images were evaluated afterwards, and the total number of adhering cells in 10 random ﬁelds of view (0.127 mm^2^) was calculated.

### Time-lapse microscopy of adhering neutrophils

For live cell imaging, HL-60 cells untreated or treated with KPL-1 were seeded on ICAM-1-precoated coverslip, then placed in a temperature-controlled incubator at 37°C and recorded under a Nikon microscope (Eclipse 80i). Time-lapse images were captured at 30s intervals with a charge-coupled device camera. The recorded data were used for analyses of HL-60 cell adhesion.

### Flow cytometry

HL-60 cells were treated as described in cell stimulation and ﬁxed with 4% paraformaldehyde. For the ﬂow cytometric analyses, cells were incubated with anti-CD18, anti-CD11a, anti-CD11b, and anti-αL/M/X/β2, and then cells were incubated with FITC-conjugated anti-mouse antibody at 22°C for 30 min, washed, and analyzed on a ﬂow cytometer (Beckman Coulter). For inhibition experiments, HL-60 cells were preincubated with MβCD (10 mM), Piceatannol (10 μM) or equal volume of DMSO/PBS at 37°C for 30 min before stimulation with mIgG or KPL1 and F(ab’)2. To assess the ICAM-1-IgG binding by ﬂow cytometry, cells were incubated with 40 µg/ml ICAM-1-IgG for 30 min on ice, washed, and then incubated with FITC-labeled anti-human IgG for 30 min on ice. 

### Immunoﬂuorescence microscopy

Cells were stimulated as above, later ﬁxed with 4% paraformaldehyde at room temperature for 10 min. Then the cells were treated with 3% BSA for 30 min and stained with anti-CD18 antibody and TRITC-conjugated anti-mouse IgG. The labeled cells were observed with a confocal fluorescence microscope (FluoView FV1000, Olympus). To stain GM1 ganglioside, the cells were treated with Alexa Fluor 488-conjugated cholera toxin B subunit (2 μg/ml) at 4°C for 30 min, followed by ﬁxation of the cells with 4% paraformaldehyde for 10 min. For inhibition experiments, HL-60 cells were preincubated with Piceatannol (10 µM), MβCD (10 mM), or equal volume of DMSO/PBS at 37°C for 30 min before stimulation with KPL1 and F(ab’)2. The relative ﬂuorescence intensity and the particle size was respectively calculated using Image J software.

### Detergent-resistant cell lysate fractions

Detergent-insoluble cell lysate fractions were isolated on discontinuous sucrose gradients [29]. Brieﬂy, cells (0.5-2×10^8^) were washed and lysed in 1 ml lysis buffer for 20 min on ice. Lysis buffer composition was as follows: 25 mM Tris-HCl, pH 7.6, 150 mM NaCl, 5 mM EDTA, 0.5% Brij 58 supplemented with protease inhibitors (2 μg/ml aprotinin, 10 μg/ml PMSF, 2 μg/ml leupeptin) and phosphatase inhibitors (1 mM sodium orthovanadate, 30 mM disodium pyrophosphate, and 10 mM glycerophosphate). Cell lysates were homogenized with 20 strokes of a loose-ﬁtting Dounce homogenizer and gently mixed for 10 min on ice with an equal volume of 80% (wt/vol) sucrose in MNE buffer (25 mM MES, pH 6.5, 150 mM NaCl, 5 mM EDTA). Two layers of 30% (2 ml) and 5% (1 ml) sucrose in MNE buffer were added successively. The discontinuous gradient was spun for 18 h at 200 000g at 4°C in Beckman SW55TI rotors. Fractions collected from the gradient top were used immediately or kept frozen at -20°C until use.

### Cholera toxin assay

Detergent-insoluble cell lysate fractions collected from sucrose gradients were evaluated for the presence of lipid rafts using a cholera toxin binding assay [30]. Brieﬂy, 2 μl of each fraction was dot blotted onto Trans-Blot Transfer MediumPure Nitrocellulose Membrane (0.45 μm; Bio-Rad Laboratories, Reinach, Switzerland). Membranes were then dried for 10 min, blocked for 1 h at room temperature with 5% milk serum albumin in TBS-0.1% Tween 20 (vol/vol), and incubated for 1 h with HRP-conjugated cholera toxin (0.084 ng/mL). Cholera toxin binding was revealed by chemiluminescence.

### Cholesterol depletion and repletion

Cells were treated with MβCD for 30 min at 37°C. The cell viability was assessed by trypan blue exclusion and staining with propidiumiodide. To replenish membrane cholesterol, the MβCD treated cells were incubated with 25 mM cholesterol for 30 min at 37°C.

### Immunoprecipitation and immunoblotting

HL-60 cells (1×10^7^ per sample) were stimulated as explained above. The cells were then lysed in the buffer (50 mM Tris -HCl, pH 7.5, 150 mM NaCl, 1 mM EDTA, 1 mM EGTA, 1% NP-40, 2.5 mM sodium pyrophosphate, 1 mM NaF, 1 mM Na3VO4, 1 mM β-glycerophosphate, and 20 μg/ml aprotin/leupeptin/PMSF). After incubation on ice for 15 min, the lysates were centrifuged at 12,000×g for 25 min. The supernatants were incubated with the indicated antibodies at 4°C for 2 h, and then 20 μl of protein A/G-Sepharose beads (50% slurry) was added. After incubation for another 1 h at 4°C, the immunoprecipitates were washed three times with lysis buffer and resolved by SDS-PAGE. Proteins were transferred to nitrocellulose membranes. The membranes were incubated with 5% nonfat milk in TBST (20 mM Tris -HCl, pH7.5, 50 mM NaCl, 0.05% Tween 20), and then with the indicated primary antibodies and the HRP-conjugated secondary antibodies at 37°C for 1 h respectively. Chemiluminescent detection was performed by using ECL Plus Western blotting reagents. For inhibition experiments, HL-60 cells were preincubated with Piceatannol (10 μM), MβCD (10 mM) or equal volume of DMSO at 37°C for 30 min before stimulation with mIgG or KPL1 and F(ab’)2.

### Statistical analysis

Quantitative data are expressed as mean ± SD. The statistical differences between the means were determined by one-way ANOVA.

## Results

### PSGL-1 ligation primes β2 integrin dependent HL-60 adhesion to ICAM-1

**Figure 1:**
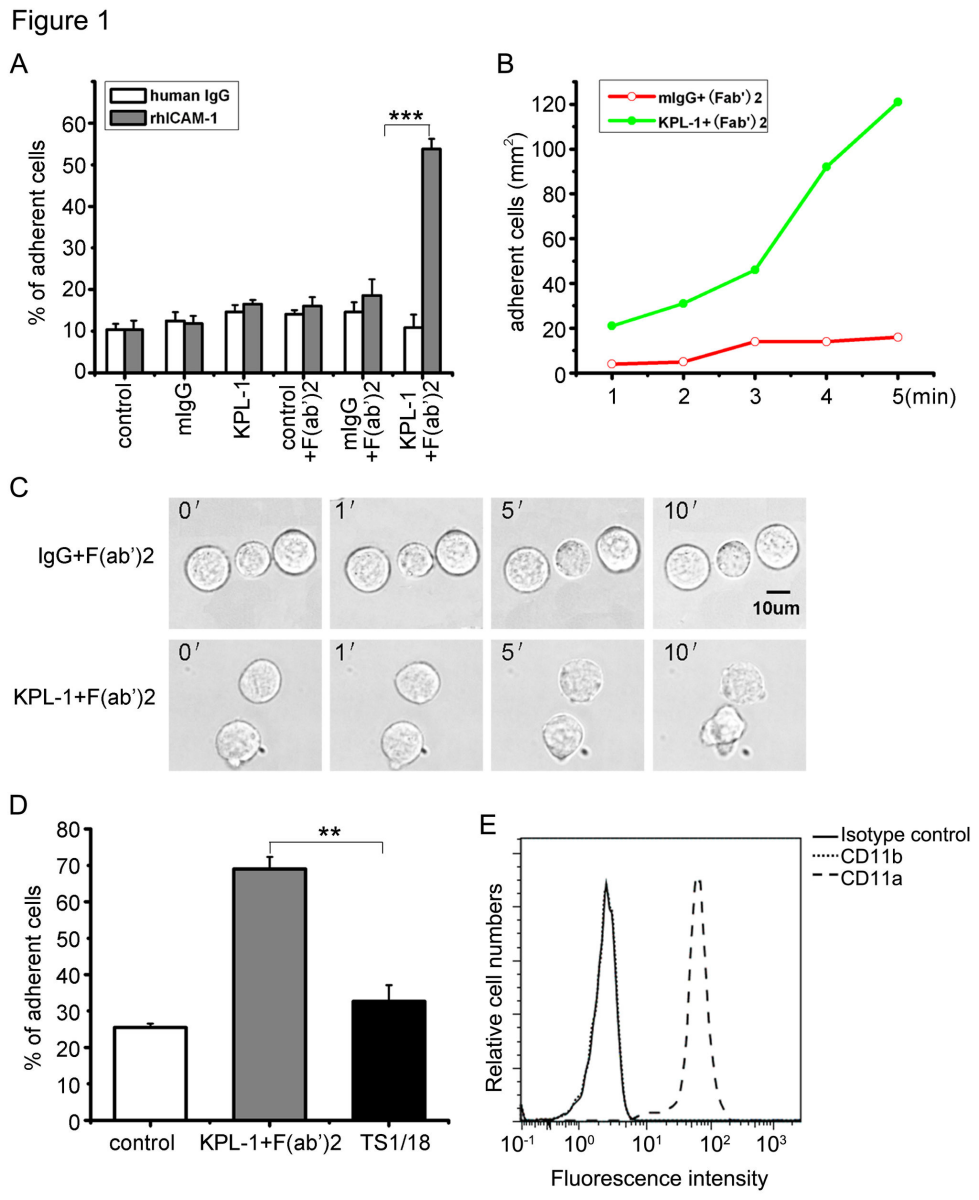
PSGL-1 ligation prime adhesion of HL-60 cells to ICAM-1. A, HL-60 cells were preincubated with calcein-acetoxymethyl ester at 37°C for 30 min. The labeled cells were incubated with or without KPL-1 or control mIgG, washed, and then incubated with or without a secondary goat F(ab’) 2 anti-mouse IgG. The cells were added to 96-well plates coated with rh-ICAM-1 or human IgG. The percentage of sustained adhesion cells was calculated by dividing the ﬂuorescence of plates after washing by the ﬂuorescence measured before washing and multiplied by 100. B, HL-60 cells were infused into ICAM-1-coated parallel-plate chamber at a shear stress of 1 dyn/cm^2^. The number of cells bound to the Polystrene Petridishes was determined. C. Time-lapsed photomicrographs (20 × objective) of HL-60 cells, which were unstimulated or stimulated with KPL-1 were obtained for 10 min, as described in Materials and Methods, and subjected to quantitative analyses of cell adhesion. Original scale bar = 10 µm. D, HL-60 cells were preincubated with TS1/18 (a leukocyte adhesion-blocking antibody to β2 subunit), before addition to the ICAM-1-coated wells, and the percentage of sustained adhesion cells was calculated as described above. Results are presented as means ± SD of values from three independent experiments. E. HL-60 cells were incubated with β2 antibody CD11a or CD11b followed by FITC-conjugated secondary antibody and analyzed by ﬂow cytometry. Results are representative of three independent experiments. All Statistical differences were determined by One-way ANOVA. **, p < 0.01, ***, p < 0.001.

To investigate whether PSGL-1 can activate β2 integrin, we analyzed the changes in adhesion of HL-60 cells to immobilized ICAM-1. Under static condition, PSGL-1 ligation with KPL1 alone could not induce HL-60 cell adhesion to immobilized ICAM-1. However, when PSGL-1 was ligated with KPL1 and subsequent secondary antibody F(ab’)2 at 37°C for 10 min, the cell adhesion significantly increased compared with the cells incubated with mIgG plus F(ab’)2 (Figure 1A). No signiﬁcant increase was observed in the HL-60 cells binding to control human IgG after ligation. Flow chamber assay was used to evaluate the effect of PSGL-1 ligation on the cell adhesion to ICAM-1 under ﬂow conditions. When HL-60 cells were perfused over the rhICAM-1-Fc monolayer via a syringe pump at a shear stress of 1 dyn/cm^2^, PSGL-1 ligation enhanced the cell binding to ICAM-1, reaching a 5-fold increase in the number of bound cells compared with the non-ligated cells at 5 min after the begining of recording (Figure 1B). We quantitatively investigated the effect of PSGL-1 ligation on HL-60 cell adhesion and morphological changes using time-lapsed videomicroscopy to record HL-60 adhesion in ICAM-1 coated plate for 10 min (Figure 1C). We found that PSGL-1 ligated HL-60 cells, not the control cells, exhibited a hypermotile phenotype, and spread on ICAM-1-coated coverslips.

To verify the roles of β2 integrin in HL-60 cells binding to ICAM-1, we performed antibody-blocking experiments. When HL-60 cells were preincubated with TS1/18 (the anti-β2 integrin blocking antibody), the percentage of adherent cells was dramatically reduced (Figure 1D). To clarify which β2 integrin(s) is/are involved in the PSGL-1 ligation-dependent β2 integrin mediated cell adhesion, β2 integrin was analyzed by flow cytometry using anti-CD11a, which reacts with LFA-1, and anti-CD11b, which recognises Mac-1, respectively. As shown in Figure 1E, HL-60 cell only expressed LFA-1 integrin. These results demonstrate that PSGL-1 ligation is required for LFA-1 integrin dependent HL-60 cell adhesion.

### PSGL-1 ligation induces β2-integrin clustering

**Figure 2:**
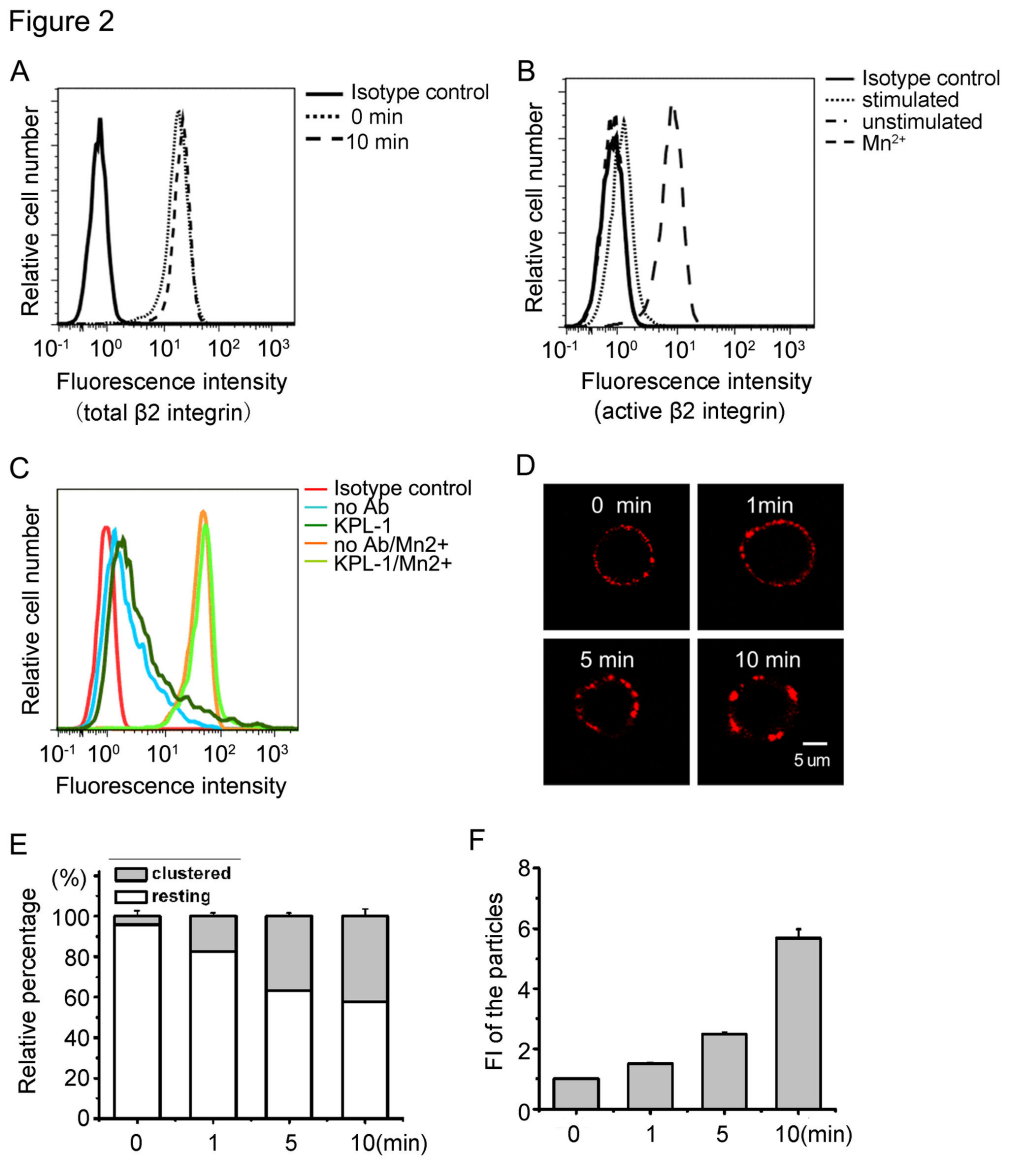
Effects of PSGL-1 ligation on β2 integrin activation. A, B, C. HL-60 cells were incubated with or without KPL-1 and a secondary antibody. Then the cells were incubated with β2 antibody IB4 (total β2), αL/M/X/β2 (active β2) or ICAM-I-IgG followed by FITC-conjugated secondary antibody and analyzed by ﬂow cytometry. Results are representative of three independent experiments. D, Cells were ligated with antibodies for indicated times and stained with IB4 and TRITC-conjugated secondary antibody. After washing, cells were observed under a confocal fluorescence microscope and typical cells shown were chosen randomly. E, HL-60 cells were counted in 5–10 fields (20 ×) and the percentages were calculated by dividing the number of resting or clustered cells by the total cells. Data were obtained from >25 cells in each field. F. The mean ± SD values of ﬂuorescence intensities of the particles are presented for mAb IB4-stained HL-60 cells. These were quantified by image analyses using the function of particle analysis in Image J program. Bars represent mean ± SD of three independent experiments.

To efficiently bind their ligands, integrins must be appropriately activated. Integrin activation is determined by its conformational changes, clustering at the cell surface and alterations in subcellular localization [15,16]. To clarify the mechanisms involved in β2 integrin activation in response to PSGL-1 ligation, β2 integrin activation was analyzed by flow cytometry using IB4 which reacts with total β2 integrin and/or αL/M/X/β2 which recognizes the activation-dependent epitope of β2 integrin. As shown in Figure 2A, no signiﬁcant alteration in β2 integrin expression on the cell surface was observed after PSGL-1 ligation with KPL1 plus F(ab’)2 for 10 min at 37°C. Treatment of the cells with Mn^2+^ resulted in the up-regulation of the active β2 integrin, whereas the PSGL-1 ligation-stimulated cells did not induce the up-regulation (Figure 2B). We also found that soluble ICAM-1 bound poorly to nonstimulated cells or the cells stimulated with PSGL-1 ligation. However, when the cells were stimulated with Mn^2+^, binding of soluble ICAM-1 was detected (Figure 2C).

Following PSGL-1 ligation for indicated times (0 min, 1 min, 5 min, 10 min), there was a rapid and extensive clustering of β2 integrin on the HL-60 cells surface. With PSGL-1 ligation, the β2 integrin clustering were larger (Figure 2D). The ratio of clustered cells to total cells was significantly increased with PSGL-1 ligation (Figure 2E). Similarly, the particle ﬂuorescent intensities as shown in Figure 2F also increased. Here, we find that PSGL-1 ligation can induce the clustering of β2 integrin on the cell surface, suggesting that the increase in β2 integrin avidity is responsible for the enhanced adhesion to ICAM-1.

### Lipid raft is required for PSGL-1 ligation induced HL-60 cell adhesion to ICAM-1

**Figure 3:**
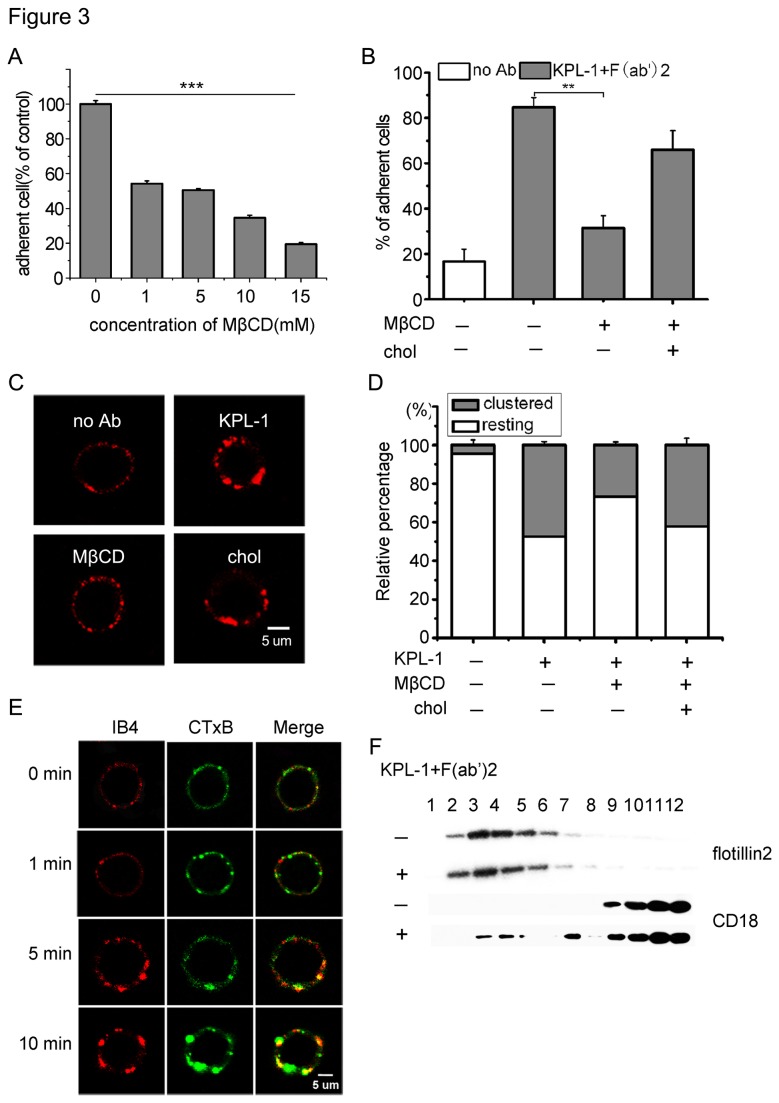
Lipid raft is involved in PSGL-1 ligation induced activation of β2 integrin. A, The PSGL-1 ligated HL-60 cells were pretreated with MβCD for 30 min at indicated concentrations, and then the cells were allowed to bind to the plates coated with ICAM-1. Cells adherent to rh-ICAM-1 were expressed as a percentage of the untreated control. Data are representative of three independent experiments and all Statistical differences were compared with control cells (without treatment). B, Cells were pretreated with MβCD (10 mM) or replenished with cholesterol (chol) after MβCD treatment, then ligated. C, HL-60 cells were treated as in B, and the treated cells were stained with IB4 for β2 integrin followed by TRITC-conjugated secondary antibody, then the cells were analysed under confocal microscope. D, The number of resting or clustered cells was counted and the percentages were calculated as described in Figure 3C. E, Cells were ligated for indicated times, and then the cells were stained with Alexa Fluor 488 conjugated-Ctx-B and IB4 Plus TRITC-conjugated secondary antibody. The merged images demonstrate the colocalization (yellow). Original scale bar = 5 μm. F, HL-60 cells were ligated or not, then cells were lysed and lipid rafts were isolated by fractionation. β2 integrin in fractions (1 to 12) was detected by western blotting. Flotillin-2 was employed as lipid raft marker. Data are representative of three independent experiments. Bars represent mean ± SD of three independent experiments. All Statistical differences were determined by One-way ANOVA. **, P < 0.01, ***, P < 0.001.

In a variety of cell types, lipid raft is required for membrane receptor to transduce signals. An earlier study has shown that lipid raft is important for the PSGL-1 mediated leukocyte rolling [28]. To test whether lipid raft was involved in PSGL-1 ligation-induced and β2 integrin dependent HL-60 cells adhesion to ICAM-1, we exposed HL-60 cells to the cholesterol depleting drug MβCD. As shown in Figure 3A, MβCD treatment inhibited HL-60 cell adhesion on ICAM-1 in a dose-dependent manner. Cholesterol replenishment greatly restored the decreased cells adhesion induced by MβCD treatment (Figure 3B). Moreover, we found that the expression of β2 integrin and PSGL-1 were not affected in the MβCD treated cells (Fig. *A* in File *S1*). Our results above indicate that lipid rafts integrity is required for PSGL-1 ligation induced and β2 integrin dependent HL-60 cell adhesion. We further investigated the effects of lipid rafts on the distribution of β2 integrin. Following MβCD treatment, the clustering of β2 integrin on the PSGL-1 ligation-stimulated cells was reduced to the levels of resting cells. However, cholesterol replenishment obviously abolished the inhibitory effect of MβCD on the clustering of β2 integrin (Figure 3C). The ratio of the number of resting or clustered cells to the total cells is shown in Figure 3D. These results indicate that lipid rafts play a crucial role in PSGL-1 ligation induced β2 integrin clustering.

To determine whether β2 integrin is associated with lipid rafts, we examined the colocalization of β2 integrin and lipid raft marker GM1 by confocal microscopy. As illustrated in Figure 3*E*, β2 integrin and lipid raft were evenly distributed over the entire cell surface in untreated cells. Upon PSGL-1 ligation, β2 integrin and lipid rafts clustered and colocalized. To conﬁrm β2 integrin is associated with lipid raft, the cells were unligated or ligated with KPL-1 and then assayed for the association of β2 integrin and lipid raft using the sucrose density gradient centrifugation. The low density fractions 3 to 5 represent the detergent-insoluble membrane components including lipid rafts, while the detergent-soluble proteins are present in the high-density fractions 9 to 12. The localization of lipid rafts in fractions 3 to 5 was confirmed by identifying the lipid raft marker flotillin-2 (Figure 3F). All β2 integrin in the unligated cells were localized in the fractions 9 to 12. Upon ligation with KPL-1, a portion of β2 integrin was observed in the pooled fractions 3 to 5, suggesting a translocation of β2 integrin to lipid rafts. These results suggest that β2 integrin is associated with lipid rafts in PSGL-1 ligated cells, but not in resting cells. 

### The integrity of lipid raft is required for PSGL-1 distribution

**Figure 4:**
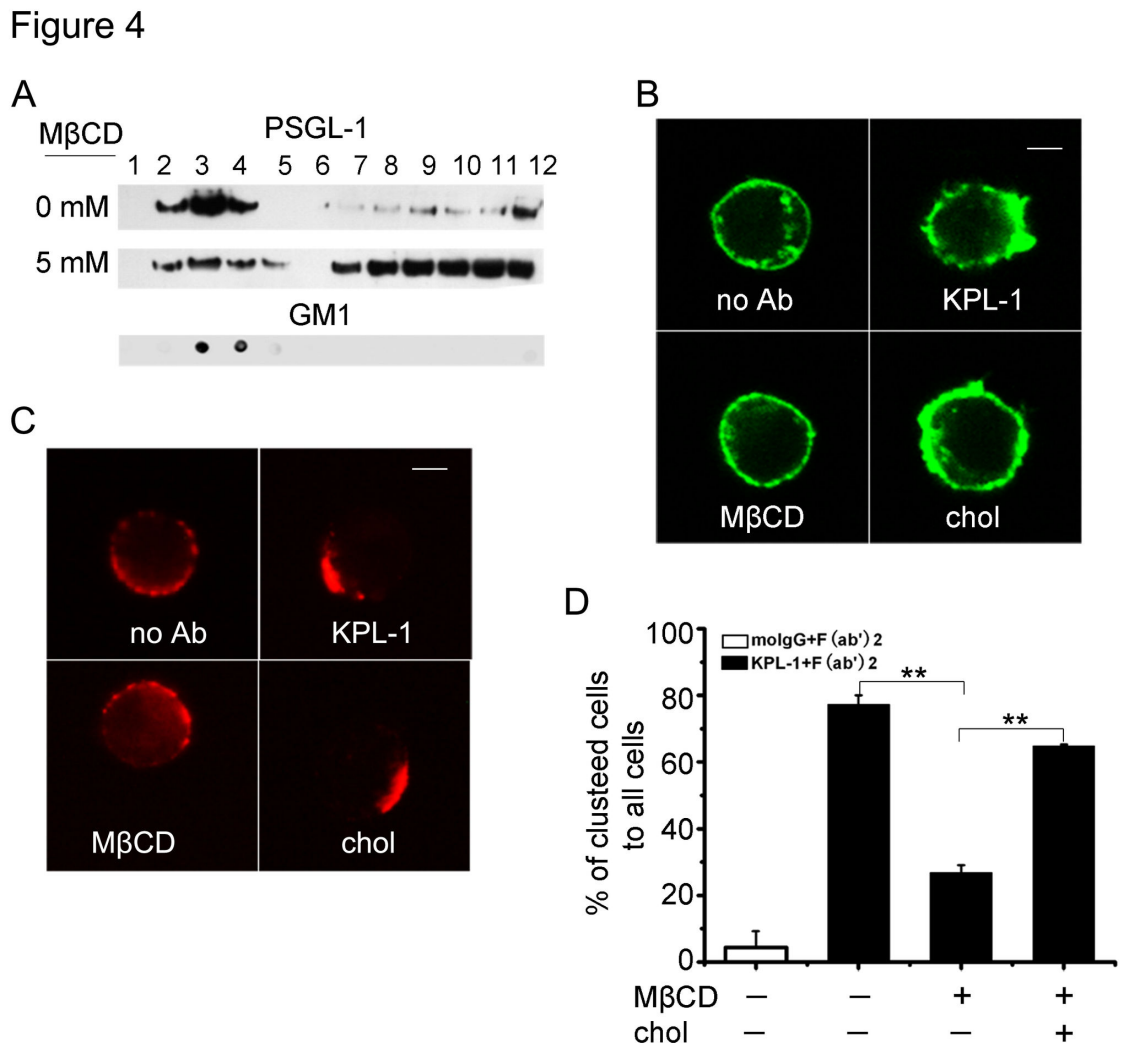
Lipid raft affects PSGL-1 distribution. A, Cell lysate fractions, separated by the sucrose density gradient centrifugation, were evaluated by immunoblotting for PSGL-1. Fractions were isolated from resting HL-60 cells or the cell treated with 10 mM MβCD. GM1 was used as lipid raft marker. B, HL-60 cells were ligated or not, or the cells were pretreated with MβCD before ligation, or reincubated with chol (cholesterol). F-actin was visualized by staining with FITC-conjugated phalloidin. C, Cells were treated as in B, and then the treated cells were labeled with KPL-1 and TRITC-conjugated secondary antibodies. Pictures shown were typical ones under fluorescence microscope. D, The ratio of clustered HL-60 cells treated as in C was calculated. Data are representative of three independent experiments. Bars represent mean ± SD of three independent experiments. All statistical differences were determined by One-way ANOVA. **, P < 0.01.

It is well established that PSGL-1 is detectable in membrane lipid rafts [27,28], however, the requirements for its localization in these membrane microdomains and the biological role of this localization are not yet well known. This prompted us to examine whether lipid raft is essential for PSGL-1 functions. We first examined PSGL-1 distribution in lipid raft. As shown in Figure 4A, PSGL-1 were detected in the fractions 3 to 5 from HL-60 cell lysates, and the localization of lipid rafts in fractions 3 to 5 was confirmed by a dot blot analysis identifying the lipid raft marker, glycosphingolipid GM1. Interestingly, the distribution of PSGL-1 in lipid raft was decreased in MβCD treated HL-60 cells (Figure 4A, intermediate panel, lanes 3 to 5). We further examined the effect of lipid raft on PSGL-1 induced downstream signaling. As our previous work have demonstrated that PSGL-1 ligation leads to cytoskeletal rearrangement [31], we analyzed the cytoskeletal changes by immunofluorescence assay under a confocal microscope. We found that ligation of HL-60 cells with PSGL-1 antibody induced F-actin redistribution. Preincubation of HL-60 cells with 10 mM MβCD inhibited the F-actin redistribution to the similar level of the resting cells, and the inhibitory effect was reversed by replenishing cholesterol implying the direct involvement of lipid raft in PSGL-1 mediated signaling pathway (Figure 4B). 

We next examined the distribution of PSGL-1 on the cell surface following its ligation. In resting cells, PSGL-1 evenly distributed over the entire cell surface with only a few small clusters. Upon ligation with antibody, PSGL-1 clustered on most of the cells, which formed a cap-like structure. Conversely PSGL-1 clustering was dramatically inhibited in the cells exposed to MβCD. Cholesterol replenishment obviously restored the decreased clustering of PSGL-1in MβCD treated cells (Figure 4C), and the ratio of the clustered cells to the total cells is shown in Figure 4D. These data demonstrate that the integrity of lipid raft is essential for PSGL-1 distribution.

### Syk localizes to lipid rafts upon stimulation of HL-60 cells with PSGL-1 ligation

**Figure 5:**
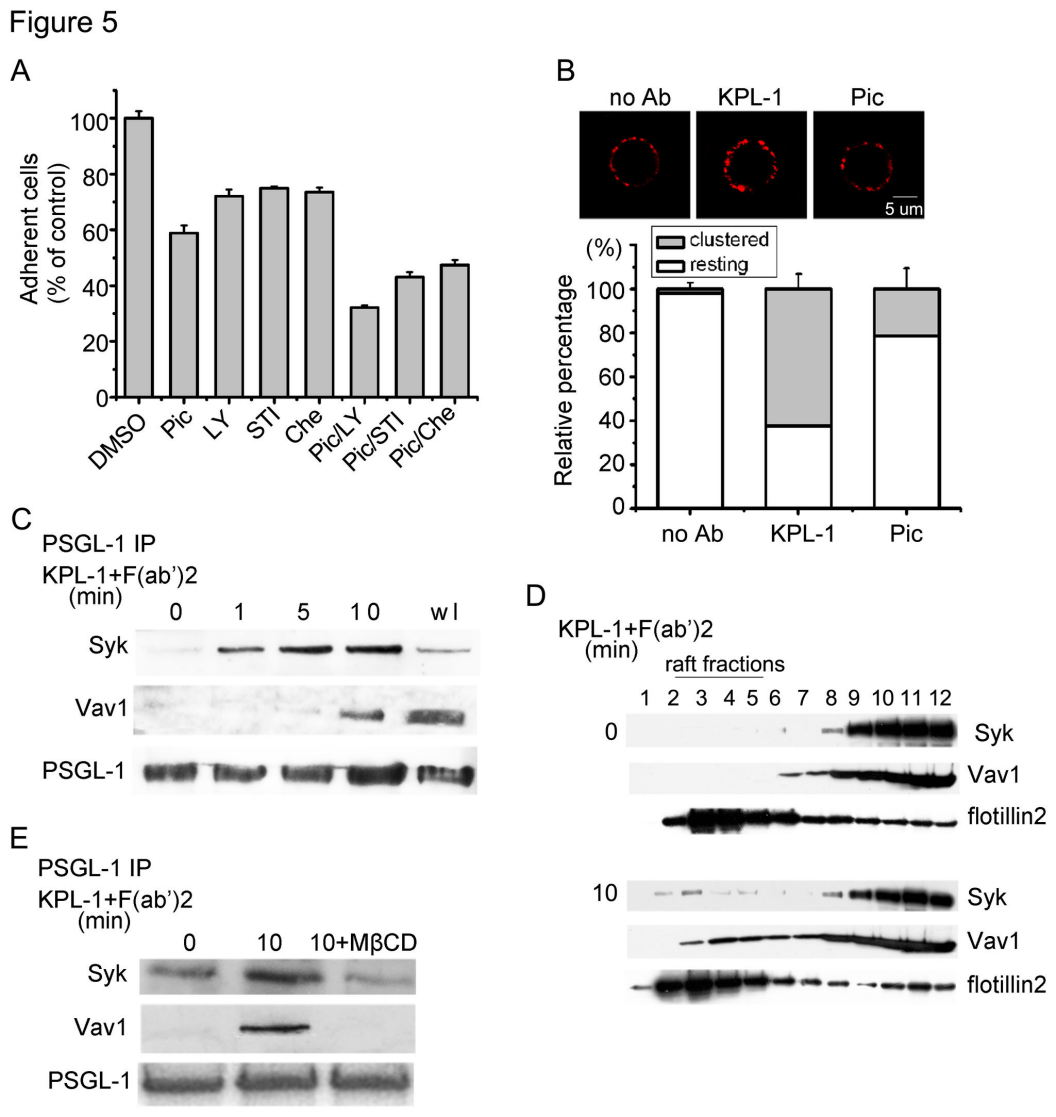
Syk and Vav1 translocate to lipid raft in PSGL-1 ligated HL-60 cell. A, HL-60 cells were respectively pretreated with 10 μM Piceatannol (Pic), 10 μM STI571 (STI), 50 μM LY294002 (LY), 20 μM Chelerythrine chloride (Che) or DMSO, and then ligated with KPL-1. Adhesion events were visualized by using a ﬂuorescence microplate reader. B, Cells were incubated with DMSO or Pic before antibody ligation, and the surface distribution of β2 integrin was observed under a confocal fluorescence microscope. Each picture is a representative of three independent experiments, and the ratio of clustered cells to total cells was calculated. Original scale bar = 5 μm. C, HL-60 cells were ligated with KPL-1 for indicated times. Then the cells were lysed and the supernatants were incubated with PSGL-1 antibody for immunoprecipitation as indicated. PSGL-1, Syk, and Vav1 were detected with corresponding antibodies by western blotting (wl: whole lysis of HL-60 cells). D, Cells were ligated or not, lysed in Brij 58 lysis buffer, and the fraction was subjected to sucrose density gradient centrifugation. The fractions were analyzed by SDS-PAGE and Western blotting with respective mAbs directed to Syk, Vav1, and Flotillin-2. E, Cells were incubated with PBS or MβCD before indicated antibody ligation, and the PSGL-1 immunoprecipitated complex was analysed respectively by Syk antibody and Vav1 antibody. Data are representative of three independent experiments. Bars represent mean ± SD of three independent experiments. All statistical differences were determined by One-way ANOVA. **, P < 0.01.

To clarify the PSGL-1-mediated signaling pathways leading to the enhanced cell binding to ICAM-1, we ﬁrst tested the effect of various kinase inhibitors on PSGL-1 ligation induced HL-60 cell adhesion. The result showed that the Piceatannol (a Syk inhibitor) pretreatment inhibited cell adhesion by 43%. In contrast, treatment of the cells with LY294002, a speciﬁc PI3K inhibitor, STI571, a c-Abl inhibitor, and Chelerythrine chloride, a PKC inhibitor moderately inhibited the cell adhesion compared to Piceatannol treatment (Figure 5A). The combined treatment of the cells with Piceatannol and LY294002 or STI571 or Chelerythrine chloride, markedly inhibited cell adhesion. Therefore Syk kinase was crucial for PSGL-1 ligation induced HL-60 cell adhesion. We found that Piceatannol treatment also inhibited β2 integrin clustering (Figure 5B), but the expression of β2 integrin was not affected in the Piceatannol treated cells (Figure *B* in File *S1*). These results suggest that Syk activation is essential for β2 integrin activation in PSGL-1 ligated HL-60 cells.

It has been reported that Syk is required for PSGL-1-dependent rolling on selectins [28], and Vav1 is one of the important substrates of Syk kinase [32]. Our previous work has showed that Vav1 is involved in PSGL-1 outside-in signaling in regulating neutrophil rolling [31]. Thus, we presumed that Syk kinase and Vav1 were involved in the same pathway downstream of PSGL-1 engagement. We ligated HL-60 cells for indicated times (0 min, 1 min, 5 min, 10 min) and lysed the cells, and then the lysates were used for coimmunoprecipitation assay. As shown in Figure 5C, after PSGL-1 ligation, Syk kinase and Vav1 were present in the PSGL-1-immunoprecipitated complexes in a time-dependent manner. 

In lymphocytes, the activation and subsequent lipid raft localization of either BCR or TCR is accompanied by the recruitment of key proximal signaling molecules such as, the tyrosine kinase Syk or ZAP-70 and the phospholipases PLCγ2 or PLCγ1, to the lipid raft microdomains of B or T cells [33]. We examined whether PSGL-1 signal complex was also translocated to lipid rafts when HL-60 cells were stimulated with PSGL-1 ligation. As shown in Figure 5D, there was no detectable Syk and Vav1 in the lipid rafts (fractions 3 to 5 as determined by the presence of the lipid raft-enriched flotillin-2) of HL-60 cells prior to any stimulation. However, PSGL-1 ligation induced a small amount of Syk and Vav1 translocated to the lipid raft fractions of HL-60 cells, suggesting that the signal molecules were recruited to special cell membrane microdomains upon PSGL-1 ligation. 

To assess the significance of lipid raft in PSGL-1 signal pathway, we pretreated HL-60 cells with MβCD to examine whether the downstream signal events would be affected when the localization of PSGL-1 to lipid raft was disrupted. We showed in Figure 5E that Syk and Vav1 were associated with PSGL-1 in stimulated cells. However, these associations were obviously disrupted in MβCD treated HL-60 cells. These results suggest that the integrity of lipid raft is crucial for the PSGL-1-Syk-Vav1 signaling pathway.

## Discussion

Considering the sequence of the events, that is, selectin-mediated rolling occurs ﬁrst and integrin-mediated adhesion occurs afterward. Selectins appear to play a role in “priming” leukocytes for optimal activation of integrin [1-3]. Current evidence supports that PSGL-1 is involved in the integrin-dependent adhesion in different leukocyte types, including neutrophils, monocytes, and lymphocytes [17,34,35]. It has also been reported that lipid raft is involved in the regulation of PSGL-1 function as rolling receptor [17,28]. However, it is not yet known if lipid raft is involved in PSGL-1 regulated integrin activation and the mechanism has not been well identiﬁed. Here, we report an essential role of lipid raft in regulating PSGL-1 ligation-induced β2 integrin-dependent HL-60 cell adhesion. The adhesion assay and immunofluorescence observation indicated that treatment with cholesterol-depleting agent MβCD signiﬁcantly impacted β2 integrin clustering at the cell membrane and resulted in a markedly defect in HL-60 cell adhesion behaviors (Figure 3A–3D), and we also found that β2 integrin was associated with lipid raft (Figure 3E, 3F). Several reports have implicated the role of lipid raft in regulating integrin function, receptor signaling and cytoskeleton dynamics [26,36]. On the basis of our ﬁndings, we suggest that lipid raft, by organizing PSGL-1 signals to activate integrin, plays a crucial role in β2 integrin-dependent HL-60 cells adhesion. Thus, our results extend the previous understanding of how PSGL-1 regulates integrin activation.

Our ﬁndings led us to seek out the downstream effectors and reveal the possible mechanism by which lipid raft regulates β2 integrin-dependent HL-60 adhesion. PSGL-1/ligand bindings have been reported to stimulate kinases such as Src, c-Abl, p38 MAPK and PI3K [8,10,31,37]. Previous study demonstrated that PSGL-1 associates with the actin-linking protein moesin, which acts as an adaptor molecule to support PSGL-1 interaction with Syk [38]. Here, we found that PSGL-1 ligation with antibody induced Syk and Vav1 recruitment to lipid rafts (Figure 5D) and that MβCD treatment strongly inhibited the relationship between PSGL-1 and these downstream molecules (Figure 5E). Although in the past decade, PSGL-1 functions as a signal transduction receptor to trigger a series of signal events have been well identiﬁed, however, it is not yet known if lipid raft regulates all the intracellular signal events in PSGL-1 engaged leukocyte. Here, we only report that Syk-Vav1 is recruited to lipid raft after PSGL-1 ligation which may initiate the downstream signaling. 

Lipid rafts are thought to be required for cell functions, including directed mobility and capping of membrane proteins, receptor-mediated signaling and membrane trafficking [18,23]. Lipid rafts are dispersed when cell cholesterol is extracted [39]. Hence, an effect of cholesterol depletion on a particular function is usually assumed to show that lipid rafts are required for this function. Kwik et al have suggested that depletion of cholesterol reduces PIP2 levels in the plasma membrane which can regulate organization of the actin cytoskeleton in resting fibroblasts and lymphoblasts [40]. In our present work, we also found that depletion of cholesterol affect the F-actin reorganization (Figure 4B) in PSGL-1 ligated HL-60 cells. However, we did not detect any effect on F-actin organization when cholestetol was depleted in resting HL-60 cell. This result suggests that the reorganization of F-actin is a result of intracellular signal events induced by PSGL-1 ligation but not the depletion of cholesterol. We also showed that Syk, Vav1 and β2 integrin were recruitment to lipid rafts in PSGL-1 ligated cells (Figures *5D*,*3F*), suggesting that lipid raft function as signaling platform to aggregate the signal molecular and membrane proteins as discussed in many reported work [29,33,40].

T cell receptors, B cell receptors, some integrins, and other molecules [33,41,42] have been reported to be localized in cholesterol-rich microdomains. Although it is well established that PSGL-1 is located in lipid rafts [28,43,44], the relevance of this association with regard to function remains to be understood. An earlier study has shown that PSGL-1 localization in lipid rafts is required for its mediates leukocyte rolling on selectins [28]. We further showed that the disruption of lipid raft significantly inhibited the location of PSGL-1 in lipid raft and the ligation-induced redistribution of PSGL-1 (Figure 4A, 4B). Our results also showed that PSGL-1 ligation induced clustering of both PSGL-1 and β2 integrin (Figure 4B, Figure 2D). Our results also show that not only chemokines [45,46] but also PSGL-1 ligation can induce cell polarization and adhesion molecule redistribution, both of which are thought to be important for cell migration. Although the relationship between PSGL-1 and β2 integrin clustering is not clearly addressed in this study, it is likely that PSGL-1 clustering is a prerequisite for β2 integrin clustering, because the increase in β2 integrin-dependent adhesion was not induced by the incubation of the cells with KPL-1 alone, but required the secondary antibody (Figure 1A). 

**Figure 6:**
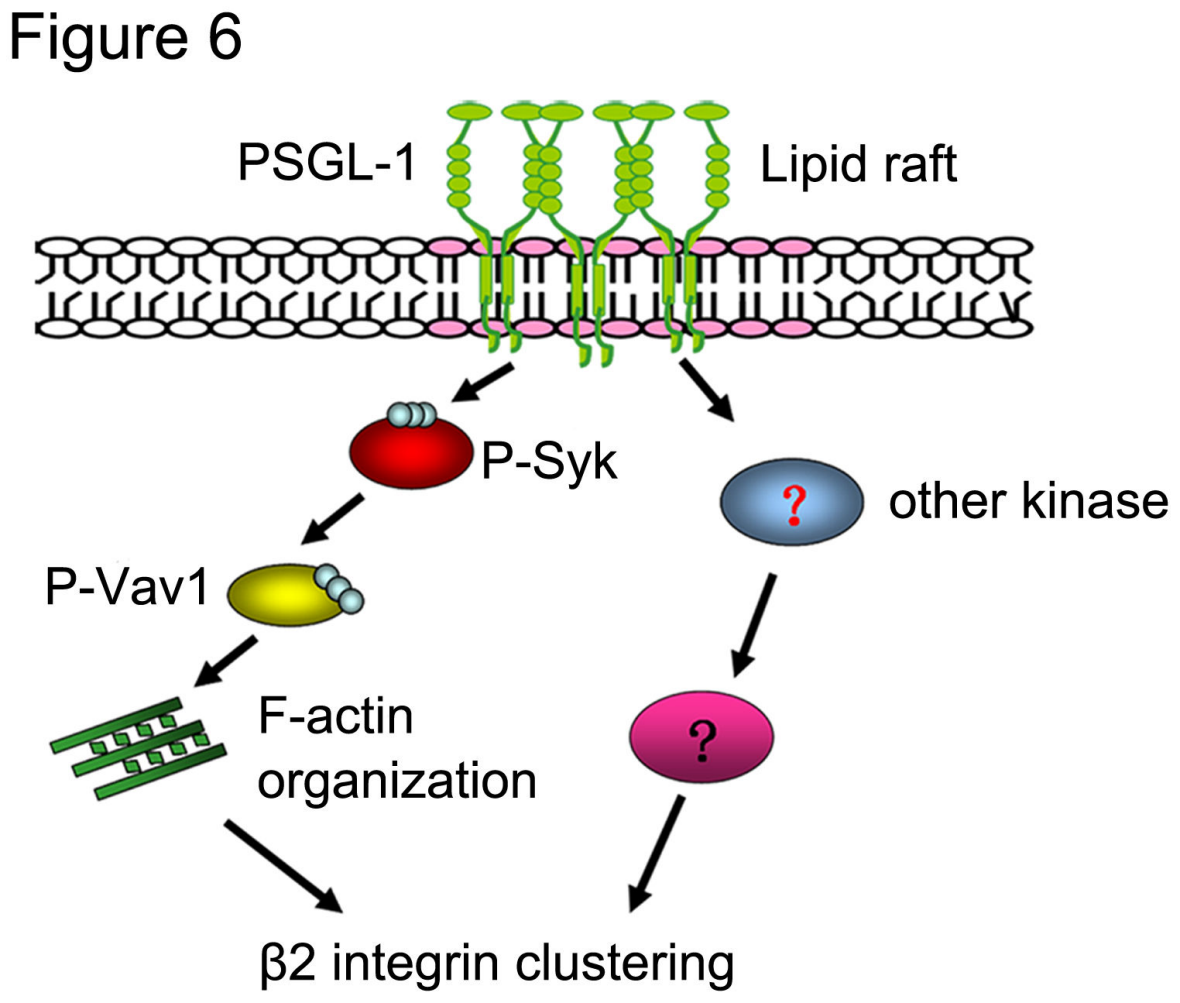
Model for PSGL-1–mediated signaling. A model of the signal transduction pathway induced by PSGL-1, which involves lipid rafts, Syk-Vav1 complex and other kinases. This signaling cascade leads to LFA-1integrin clustering that enables HL-60 cell adhere on ICAM-1.

Here, we demonstrate that lipid raft is crucial for β2 integrin activation and β2 integrin dependent adhesion in PSGL-1 ligated HL-60 cells, and the disruption of lipid raft integrity affect HL-60 cells adhesion behavior signiﬁcantly. In addition, we show that the disruption of lipid rafts affect the distribution of PSGL-1 as well as the PSGL-1 induce cellular signaling events. Furthermore, PSGL-1 ligation induces the Syk-Vav1 recruitment to lipid raft, which may ultimately regulate the organization of cytoskeleton to stabilize the β2 integrin clustering (Figure 6). To our knowledge, this is the ﬁrst evidence to demonstrate that a crucial role of lipid raft in PSGL-1 signaling regulated the integrin activation.

## Supporting Information

File S1 Figure A, MβCD treatment does not affect the expression of PSGL-1 and β2 integrin. HL-60 cells were incubated with or without MβCD (10 mM), then the cells were stained with KPL-1(left), IB4(right), followed by FITC-labeled anti-human IgG, and analyzed by ﬂow cytometry. Results are representative of three independent experiments. Figure B, Piceatannol treatment does not affect the expression of β2 integrin. HL-60 cells were incubated with or without Piceatannol (10 µM) and stained with IB4 or human IgG followed by FITC-labeled anti-human IgG, and analyzed by ﬂow cytometry. Results are representative of three independent experiments.
(DOC)
