# Supplementary material for: Lipid Raft Is Required for PSGL-1 Ligation Induced HL-60 Cell Adhesion on ICAM-1
Source: PLoS One. 2013 Dec 3;8(12):e81807. doi: 10.1371/journal.pone.0081807 (PMC3849276; doi:10.1371/journal.pone.0081807)
Supplement: File S1 — Figure A, MβCD treatment does not affect the expression of PSGL-1 and β2 integrin. HL-60 cells were incubated with or without MβCD (10 mM), then the cells were stained with KPL-1(left), IB4(right), followed by FITC-labeled anti-human IgG, and analyzed by ﬂow cytometry. Results are representative of three independent experiments. Figure B, Piceatannol treatment does not affect the expression of β2 integrin. HL-60 cells were incubated with or without Piceatannol (10 µM) and stained with IB4 or human IgG followed by FITC-labeled anti-human IgG, and analyzed by ﬂow cytometry. Results are representative of three independent experiments. (DOC) [file pone.0081807.s001.doc]

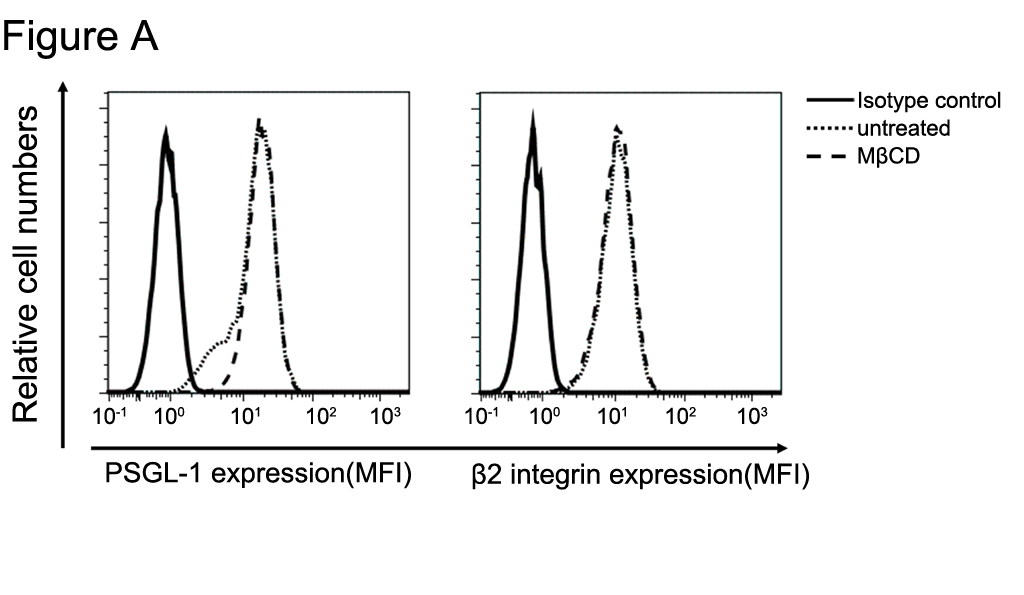


Figure A MβCD treatment does not affect the expression of β2 integrin and PSGL-1. HL-60 cells were incubated with or without MβCD (10 mM) and stained with IB4, KPL-1 or human IgG followed by FITC-labeled anti-human IgG.


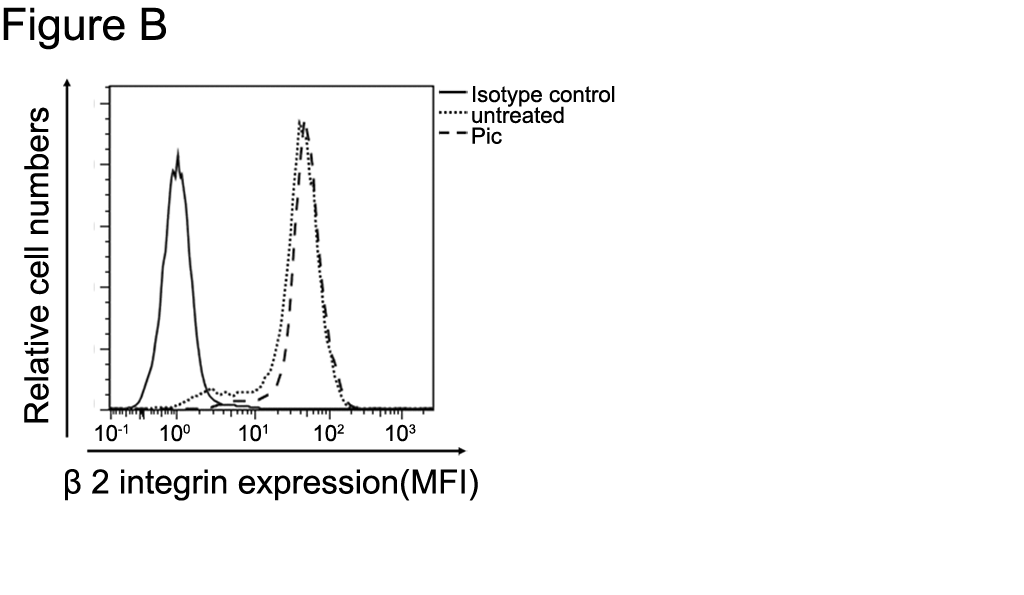


Figure B Piceatannol treatment does not affect the expression of β2 integrin. HL-60 cells were incubated with or without Piceatannol (10 µM) and stained with IB4 or human IgG followed by FITC-labeled anti-human IgG.
